# Research Supporting a Pilot Study of Metronomic Dapsone during Glioblastoma Chemoirradiation

**DOI:** 10.3390/medsci9010012

**Published:** 2021-02-16

**Authors:** Richard E. Kast

**Affiliations:** IIAIGC Study Center, Burlington, VT 05408, USA; richarderickast@gmail.com

**Keywords:** dapsone, edema, glioblastoma, interleukin-8, myeloid derived suppressor cells, neutrophils

## Abstract

This short note presents previous research data supporting a pilot study of metronomic dapsone during the entire course of glioblastoma treatment. The reviewed data indicate that neutrophils are an integral part of human glioblastoma pathophysiology, contributing to or facilitating glioblastoma growth and treatment resistance. Neutrophils collect within glioblastoma by chemotaxis along several chemokine/cytokine gradients, prominently among which is interleukin-8. Old data from dermatology research has shown that the old and inexpensive generic drug dapsone inhibits neutrophils’ chemotaxis along interleukin-8 gradients. It is on that basis that dapsone is used to treat neutrophilic dermatoses, for example, dermatitis herpetiformis, bullous pemphigoid, erlotinib-related rash, and others. The hypothesis of this paper is that dapsone will reduce glioblastomas’ neutrophil accumulations by the same mechanisms by which it reduces dermal neutrophil accumulations in the neutrophilic dermatoses. Dapsone would thereby reduce neutrophils’ contributions to glioblastoma growth. Dapsone is not an ideal drug, however. It generates methemoglobinemia that occasionally is symptomatic. This generation is reduced by concomitant use of the antacid drug cimetidine. Given the uniform lethality of glioblastoma as of 2020, the risks of dapsone 100 mg twice daily and cimetidine 400 mg twice daily is low enough to warrant a judicious pilot study.

## 1. Introduction

### 1.1. Background

As things stand as of 2020, glioblastoma (GB) is uniformly fatal within a few years of initial diagnosis. This brief note reviews accumulated research data that, taken together, indicate that adding an old antibiotic, dapsone, to current standard treatment of GB may to some degree increase survival time. The expected benefit of adding dapsone might be small but welcome. The physiologic effects and pharmacological attributes of dapsone mesh with selected aspects of GB pathophysiology in a way predicted to retard GB growth.

### 1.2. Dapsone

Dapsone is a sulfone antibiotic continuously in use worldwide since the 1950s. The two main uses of dapsone as of the end of 2020 are 1) as antibiotic and 2) to treat the neutrophilic dermatoses [1]. Table 1 lists the basic pharmacologic parameters of dapsone. The database as of 2014, supporting adjunctive dapsone use during treatment of GB was presented in our previous series of papers on this [2,3,4,5,6]. The in vitro levels of dapsone needed to demonstrate glioma cell line direct cytotoxicity are supraphysiologic and probably not relevant [2]. Further data on dapsone, relevant to GB and accruing since 2014, is reviewed here.

As a sulfone antibiotic, dapsone is used in general medicine to treat various infections—Hansen’s disease, Plasmodia, Pneumocystis, Toxoplasmodia, and others [7]. In dermatological practice, dapsone is used to treat a variety of neutrophilic dermatoses of non-microbial origin like bullous pemphigoid, dermatitis herpetiformis, urticarial dermatoses, cutaneous lupus, and others [7,8,9,10].

The collected data presented here indicates that dapsone has potential to diminish aspects of interleukin-8 (IL-8) (Section 2) and neutrophils’ (Section 3) contributions to GB growth in part by similar mechanisms as it does in promoting healing in the neutrophilic dermatoses. Vide infra.

## 2. Dapsone

### 2.1. GB and IL-8

IL-8 is integral to GB tumor cell proliferation, invasion, and vascular mimicry [11]. Details follow:

IL-8 is an 11 kDa cytokine that comprises an integral, core feature of GB’s pathophysiology [12]. In addition to the data collected and analyzed in our previous GB-related dapsone papers referenced above, recent reports confirm IL-8 as contributing to GB cells’ migration, vascular mimicry, and proliferation, as well as the established role of IL-8 as a central chemotaxic signal for neutrophil homing [11,12,13,14,15]. Autocrine IL-8 promotes motility in GB cells [16,17] and drives (among many other factors) their growth [18,19] as well as attracting neutrophils to areas of angiogenesis. Tissue from GB recurrences have increased expression of IL-8 compared to the initial tumor [18]. 

Irradiation prolongs survival in GB but also engages multiple growth-enhancing pathways, leading to more aggressive tumor on the heretofore inevitable regrowth [20,21]. As documented in other cancers, standard irradiation treatment increases IL-8 in GB: by microdialysis and stereotaxic biopsy analysis, greater irradiation-induced IL-8 increases are correlated with shorter overall survival [22]. Standard post-resection irradiation of GB also increased IL-8 in adjacent uninvolved brain tissue, thus forming a “fertilizing” tissue preparation for residual GB invasion and regrowth [22]. Such peritumoral IL-8 induced by irradiation is chemotactic to residual isolated intramural GB cells. This IL-8 increase in irradiation-exposed tissue—in both GB and normal tissues—are a major aspect driving irradiation-induced increased GB aggressiveness [21,23,24,25]. 

Greater GB expression of IL-8 receptor CXCR2 is also associated with shorter overall survival [26].

### 2.2. Dapsone, IL-8, and Neutrophils

Dapsone decreases IL-8 and interferes with IL-8 function in several settings [2,27,28,29,30,31,32]. Dapsone suppressed in vitro IL-8 production in human epidermal keratinocytes and in THP-1 leukaemic monocytes [33]. However, Bellon et al. showed no inhibition of IL-8 by dapsone in IL-1 alpha stimulated bronchial epithelial cells [34]. It is unknown if dapsone would lower IL-8 in the above-mentioned irradiation exposed brain tissue.

Necrotic islands surrounded by pseudopalisading dense sheets of hypoxic GB cells are a typical feature of GBs. Nearby these areas are dense collections of neutrophils. These hypoxic, necrotic areas stain heavily for IL-8 and are associated with shorter survival [35]. 

Elevated circulating IL-8 in GB, and the associated, consequent, neutrophil activation, as defined by CD11b expression, is associated with shorter GB survival [36]. Conclusion: IL-8 attraction of neutrophils to a growing GB is a pathophysiological parallel to the bullous and neutrophilic dermatoses. 

## 3. Dapsone, GB, and Neutrophils

Neutrophils home along an IL-8 gradient and become activated in the presence of IL-8. Neutrophils contain fully 50% of total blood vascular endothelial growth factor (VEGF) [37,38,39]. Evidence from multiple experimental and clinical studies implicate neutrophils as a growth-enhancing link in GB pathophysiology. Some of that data accumulated since 2014 is reviewed below.

Denser neutrophil infiltration in human GB is associated with a shorter overall survival [40]. During standard temozolomide chemoirradiation, circulating neutrophil decreases of ≥40% predict a longer survival than those who do not have such a dip [41].

A defining feature of human GB is the presence of necrotic areas in proximity to dense neutrophil collections and pseudopalisades. Although necrosis as cause or effect of dense neutrophil pockets has not been established, a greater degree of such necrosis within a GB is associated with shorter survival [35,42]. Necrotic tumor areas characteristic of GB also stimulate nearby GB tissue to secrete increased IL-8 [35]. 

In a study of 2249 resected gliomas of all grades, increasing neutrophil content within the tumors correlated with increased malignancy grade and shorter overall survival [43].

Five independent studies in the last few years attested to both dexamethasone use and the neutrophilia and/or lymphocytopenia caused by dexamethasone use are correlated with shorter survival and clinically meaningful immunosuppression in GB [44,45,46,47,48]. 

In a study of prednisone use in giant cell arteritis, dapsone was found to be steroid sparing [49]. Were it so in GB, this alone would be of benefit in GB. 

Promotion of malignant neovascularization and multiple other GB growth enhancing attributes of neutrophils exist, in addition to the above mentioned bearing of VEGF and IL-8 [12,49,50,51,52]. VEGF, IL-8, interleukin-1 beta (IL-1b), and other neutrophil borne cytokines, not only participate in mediation of neovascularization in GB—these also increase resistance to temozolomide. It was therefore natural and expected when in early 2019 neutrophil tumor infiltration was shown to counteract bevacizumab (Avastin^tm^) effectiveness in colon cancer [53] as it does in GB [54,55], further evidence that neutrophils indeed bring VEGF to a growing GB.

**Figure 1:**
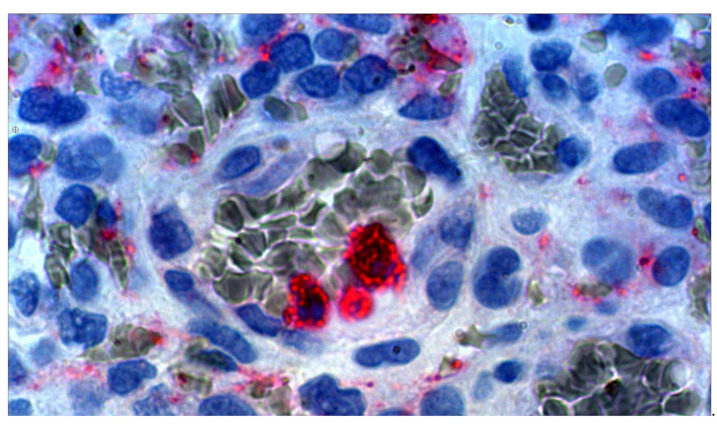
Micrograph, human GB ×100 oil immersion with electronic magnification. Anti-VEGF stained (red) neutrophils and vascular endothelial growth factor (VEGF) containing debris. With permission, thanks to A. Scheuerle, MD for this slide.

See Figure 1 showing neutrophils within or adherent to a venule wall of a human GB. Anti-VEGF immunohistochemistry stains red and red blood cells stain grey-green (thanks to A. Scheuerle, MD for this slide).

In 2020, Owen-Woods et al. demonstrated that neutrophil binding, rolling, and migrating along, and penetration between, endothelial cells is a bidirectional feedback process that is both cause and an effect of tissue edema [56], thus forming an amplifying feedback system. It is by dapsone’s potential to interrupt this amplifying feedback that dapsone may reduce the peritumoral edema characteristic of GB. Peritumoral edema is not just a mechanical problem—it is precisely the peritumoral edematous brain areas where GBs tend to recur [57].

A subset of neutrophils or neutrophil-like cells comprise the major component of myeloid derived suppressor cells present within GBs. They constitute an element of GB’s immunosuppressive environment [58,59,60,61,62,63]. The preponderance of evidence in human cancers points to a growth enhancing role in naturally occurring malignant disease—hence dapsone. Intratumoral neutrophils are a negative prognostic sign in GB and interestingly positively correlated with programmed death ligand-1 expression [64,65].

Relevant to this statement, empirically, remarkably fully 13 independent studies just between 2018 and 2020 reported the same findings—that a higher neutrophil to lymphocyte ratio—a relative or absolute neutrophilia—correlated with shorter survival in GB [66,67,68,69,70,71,72,73,74,75,76,77,78]. Few parameters in any cancer have been so oft confirmed. This correlation also applied to pretreatment, pre-steroid GB use. While not a strong effect—for example overall survival 18 months with versus 24 months without relative neutrophilia would be typical—it is nevertheless strong evidence of a certain, if limited, growth promoting role of neutrophils—one worthy of inhibition if we can do so. Absolute neutrophil count, pre-operative, and pre-treatment neutrophil count greater than 7×10^9^/L was a negative prognostic factor for OS [76].

By what specific intracellular mechanism of action dapsone reduces neutrophils’ pro-inflammation behavior has not been established fully, but inhibition of neutrophils’ myeloperoxidase seems to be important [79]. Myeloperoxidase catalyzes the reaction: H_2_O_2_ + Cl^−^ = H_2_O + OCl^−^. Dapsone, by binding to myeloperoxidase, lowers hypochlorite production. It should be noted here that GB patients have twice the level of myeloperoxidase both in plasma and in the GB tissue itself [80].

Of interest, neutrophil agglomerations form “hotspots” of concentrated serine hydrolases within GBs [81]. These are related to the neutrophil extracellular traps that are associated with GBs and worsen prognosis [78].

## 4. Dapsone Safety

Dapsone is not an ideal drug. Aplastic anemia, hypersensitivity reactions, and drug-induced hepatitis are uncommon but real risks of dapsone use. Some degree of methemoglobinemia is universal with dapsone use but this is not usually symptomatic [82]. In a study of dapsone 100 mg × 1 given over half a year to rheumatoid arthritis patients, 17% had to stop it due to side effects [83]. Considering the gravity of GB, many clinicians would consider the risk/benefit ratio to favor a pilot study of dapsone in GB.

Deps et al. found that as part of multidrug treatment (rifampin, clofazimine, dapsone) of 194 people with Hansen’s disease, 25% developed minor hemolytic anemia traceable to dapsone use (hematocrit fell 38 to 31) [82]. There have been isolated, single case reports of fatal fulminant hepatitis or agranulocytosis associated with dapsone use. Dapsone is considered safe enough for chronic use over many years in rheumatoid arthritis and lupus. Of note, dapsone reduces ESR and CRP in rheumatoid arthritis [82,83,84,85].

In their case series of dapsone use in treating various subtypes of cutaneous lupus erythematosus in 34 patients, Klebs et al. found that with an average dapsone dose of 100 mg once daily, 11 of 17 people on dapsone as monotherapy experienced full remission of cutaneous signs and symptoms [86]. One developed elevated liver enzymes with eosinophilia and elevated CRP (drug reaction with eosinophilia and systemic symptoms (DRESS syndrome), one developed digit hyperesthesia after 7 months treatment, and one person developed hemolytic anemia (hemoglobin 9.7 g/dl = 6 mM) with 72% reticulocytes. All patients developed asymptomatic methemoglobinemia [86].

People with glucose-6-phosphate dehydrogenase (G-6-PD) deficiency may be at greater risk for hematological side effects. Low level methemoglobin can give subtle signs of reduced mental function, headache, or fatigue [87].

Caution must be taken when treating people with diabetes in that HbA1c levels become unreliable during dapsone treatment [88]. Blood glucose measurement remains uninfluenced and valid.

It is important to note that in dermatitis herpetiformis, bullous pemphigoid, IgA dermatitis, and other neutrophilic dermatoses, dapsone treatment resolves the skin lesions but does not influence the auto-antigen-antibody complexes that were generating the problem.

Given that ~30% of GB patients develop seizures, an intriguing and felicitous side note—dapsone has preclinical evidence of significant anti-seizure activity [89,90,91,92] and a single open-label study in humans confirming this [93]. This, plus dapsone’s demonstrable neuroprotection in animal models [94,95,96] together constitutes indirect evidence of its ability to pass the blood-brain barrier. There is no published data on direct measurement of dapsone in CSF or brain parenchyma. The pharmacokinetics of dapsone are given in Zuidema et al [97].

## 5. Cimetidine

The widely available, inexpensive histamine receptor 2 blocking drug cimetidine 800 mg every 12 h reduces incidence and extent of dapsone related methaemoglobin, improving dapsone’s therapeutic index by reducing dapsone’s N-hydroxylation [98,99,100].

Coincidentally, cimetidine itself has extensive preclinical, and some limited clinical evidence for an anti-cancer growth effect. A comprehensive review of the potential for inhibiting cancer growth, including anti-glioblastoma aspects of cimetidine, was published in 2014 [101]. Cimetidine inhibits histamine receptor 2, the renal organic cation transporter 2, and multiple hepatic CYP enzymes, most prominently CYP1A2, CYP2D6, and CYP3A4. As a consequence, the half-life of many drugs is prolonged during cimetidine use.

## 6. Conclusions

Clinicians must weigh the not inconsiderable risks of chronic dapsone with the unproven potential benefits as outlined in this paper. Given the uniformity of fatal outcome within two years following initial diagnosis, a small pilot study of 100 mg dapsone twice daily with cimetidine 400 mg twice daily is warranted as adjuvant to current standard resection followed by temozolomide chemoirradiation.

There are many more elements driving GB growth other than IL-8 and neutrophils. But any small growth promoting element that we can deprive GB of furthers our goal of long-term control. Preclinical and clinical data indicate that dapsone may contribute, even if slightly, to that goal of reducing IL-8/neutrophil contributions.

## Figures and Tables

**Table 1:** Basic pharmacological parameters of dapsone. For references see text and Zuidema et al.

Mol. wt.	248
metabolism	N-glucuronidation, CYP 2C9, 2C19
metabolite	monoacetyl dapsone, half-life 22 h
protein bound	73%
Half-life	~20–30 h, blood
single dose Cmax	~1 mcg/mL, = ~1 mg/L = ~4 microM
Cmax, chronic	median = 16 microM = 4 mcg/mL
high blood level	5% had 28 microM = 7 mcg/mL blood
therapeutic range	0.5 to 5 mcg/mL serum [as antibiotic]
glioblastoma (GB) motility inhibition	50 microM = 12 mcg/mL, in vitro
GB growth inhibition	50 microM = 12 mcg/mL, in vitro
50% IL-8 reduction	25 microM = 6 mcg/mL
Side effects >5%	methemoglobinemia
Side effects 1–5%	hemolytic anemia
Side effects <1%	agranulocytosis, hepatitis
antibiotic use	Hansen’s disease, Pneumocystis, Toxoplasmosis, Mycobacteria, malaria
dermatology use	neutrophilic dermatoses, acne

## Data Availability

All data has been presented in the published paper.

## Abbreviations

glioblastoma	(GB)
interleukin-1 beta	(IL-1b)
interleukin-8	(IL-8)
vascular endothelial growth factor	(VEGF)
